# mHealth for Clinical Decision-Making in Sub-Saharan Africa: A Scoping Review

**DOI:** 10.2196/mhealth.7185

**Published:** 2017-03-23

**Authors:** Ibukun-Oluwa Omolade Adepoju, Bregje Joanna Antonia Albersen, Vincent De Brouwere, Jos van Roosmalen, Marjolein Zweekhorst

**Affiliations:** ^1^ Athena Institute for Research on Innovation and Communication in Health and Life Sciences Vrije Universiteit Amsterdam Amsterdam Netherlands; ^2^ Institute of Tropical Medicine Maternal and Reproductive Health Unit, Department of Public Health Antwerp Belgium; ^3^ Leiden University Medical Center Department of Obstetrics Leiden Netherlands

**Keywords:** mHealth, decision support systems, clinical, sub-Saharan Africa, clinical decision-making

## Abstract

**Background:**

In a bid to deliver quality health services in resource-poor settings, mobile health (mHealth) is increasingly being adopted. The role of mHealth in facilitating evidence-based clinical decision-making through data collection, decision algorithms, and evidence-based guidelines, for example, is established in resource-rich settings. However, the extent to which mobile clinical decision support systems (mCDSS) have been adopted specifically in resource-poor settings such as Africa and the lessons learned about their use in such settings are yet to be established.

**Objective:**

The aim of this study was to synthesize evidence on the use of mHealth for point-of-care decision support and improved quality of care by health care workers in Africa.

**Methods:**

A scoping review of 4 peer-reviewed and 1 grey literature databases was conducted. No date limits were applied, but only articles in English language were selected. Using pre-established criteria, 2 reviewers screened articles and extracted data. Articles were analyzed using Microsoft Excel and MAXQDA.

**Results:**

We retained 22 articles representing 11 different studies in 7 sub-Saharan African countries. Interventions were mainly in the domain of maternal health and ranged from simple text messaging (short message service, SMS) to complex multicomponent interventions. Although health workers are generally supportive of mCDSS and perceive them as useful, concerns about increased workload and altered workflow hinder sustainability. Facilitators and barriers to use of mCDSS include technical and infrastructural support, ownership, health system challenges, and training.

**Conclusions:**

The use of mCDSS in sub-Saharan Africa is an indication of progress in mHealth, although their effect on quality of service delivery is yet to be fully explored. Lessons learned are useful for informing future research, policy, and practice for technologically supported health care delivery, especially in resource-poor settings.

## Introduction

### Significance of mHealth

Mobile health (mHealth), defined as “the provision of health services and information via mobile technologies” (p.8; [1]), has gained widespread recognition as an innovative way of improving health care access especially in low-resource settings [2]. It is increasingly incorporated in behavioral change interventions for patients and health workers, patient monitoring, data collection, and health information systems [3-5]. With mobile subscription penetration estimated at 80% in sub-Saharan Africa [6], mHealth can potentially reduce gaps and inefficiencies in health service delivery in low-income countries [7].

In poor-resource settings such as Africa, the weak capacity of health systems is further stretched by health worker shortages, leading to the devolution of some service delivery tasks to lower cadre workers such as auxiliary nurses and community health workers. Although task shifting has been recognized for improving efficiency and access to health services, concerns exist whether lower cadre health workers are competent and equipped to effectively handle additional responsibilities [8]. The potential role of mHealth in enabling task shifting and service delivery in line with evidence-based practice is therefore important.

In high-income countries, where the health system landscape is more adapted for technological innovation than in low- and middle-income countries, knowledge on the use of technology for clinical decision-making is advanced [9-12]. A substantial body of literature presents evidence on the broad use and benefits of mHealth in low- and middle-income countries [13-18]. The extent to which mHealth has been specifically adopted in Africa to mitigate workforce shortages and maintain quality standards of care by serving as a clinical decision support system, is yet to be established. A preliminary search conducted by the first author identified only one review with a limited focus on the use of medical decision support systems in three sub-Saharan African countries [19]. As new mHealth innovations are increasingly being tested and adopted in resource-poor settings, it is necessary to comprehensively assess what has been achieved in order to inform implementers and policy makers on the effectiveness of technology in evidence-based practice.

### Objective

The aim of this study therefore was to synthesize available evidence on the use of mobile technology as an interface for improving point-of-care clinical decision-making and the quality of care in Africa.

## Methods

The 5-step framework for conducting scoping reviews as proposed by Arksey and O’Malley [20] and further developed by Levac and colleagues [21] was used as a guide in conducting this review.

### Conceptualization of Key Terms

mHealth is differentiated from the broader domain of eHealth, which includes supportive factors for the use of information and communications technology (ICT) in health such as legislation, policies, and standards. This review did not include the use of technology in health beyond mobile devices such as mobile phones, tablet computers including laptops, and personal digital assistants.

Health workers are all people engaged in the promotion, protection, or improvement of population health. The World Health Organization’s international classification of health workers [22] was used to specify health providers of interest. We were interested in health workers who are involved in making decisions on diagnosis, treatment, or other processes directly related to patient care, therefore excluding categories such as social workers that mainly provide supportive care. In more general terms, the review included doctors, nurses, midwives, associate clinicians, and lay health workers. Doctors were included in this review because the extent of decision support systems use by different health worker cadres in Africa was unknown.

Clinical decision-making involves making judgments about care provided in health service settings using information or knowledge, and can be defined as “...a contextual, continuous, and evolving process, where data are gathered, interpreted, and evaluated in order to select an evidence-based choice of action” (p.401 [23]).

We adopt the Institute of Medicines’ definition of quality of care as service delivery that increases the likelihood of desired health outcomes, is aligned to current professional knowledge and is safe, effective, patient-centered, timely, efficient, and equitable [24]. On the basis of this, we consider that quality care is the expected outcome of improved decision-making in health settings, although it may not be explicitly reported in articles.

A mobile clinical decision support system (mCDSS) in the context of this review therefore refers to any mobile electronic or computerized system that provides evidence-based information, which enhances the ability of health care providers to deliver quality care through the prevention, diagnosis, and management of health conditions. There is sufficient literature on the use of health information from electronic medical records and surveillance data to facilitate administrative decision-making or improve clinical workflow. In this review, we included interventions in which data mining or electronic medical records were not the sole component of clinical decision support.

### Building the Search Strategy

The search syntax (see Multimedia Appendix 1) was developed on PubMed using combinations and word variations of key terms for the review: mHealth, decision-making, quality of care, health care workers, and Africa, against their appropriate MeSH terms and supported by free text formats. Additional terms were included using keywords from articles of interest retrieved by a preliminary limited search on PubMed. The formula for the final search syntax was (mobile health) AND (decision-making OR quality of care) AND (health care workers) AND (Africa)

### Running the Search

In December 2015, we searched the following peer-reviewed databases: PubMed, CINAHL, Web of Science Core Collection, and Cochrane. And, the grey literature electronic database K4Health (see Multimedia Appendix 2).

Without applying date limiters, the search targeted English language articles reporting use of mHealth for clinical decision-making in African countries.Relevant articles had to be manually retrieved from the grey literature database (ie, K4Health) under the thematic heading “decision support.”

Weekly email alerts were set for all databases (except Cochrane and K4Health) to allow for additional articles that could emerge between the date of initial search and when the final decision was made on study selection for full reading. Additional articles were therefore assessed for inclusion until March 5, 2016. References were managed using EndNote X7.7 (Clarivate Analytics), a software program for managing bibliographies and citations.

### Study Selection

A total of 1158 articles were identified from running the search. After excluding duplicates, 924 articles were retained, which were screened against predetermined inclusion or exclusion criteria. Both primary and secondary (ie, literature reviews) studies were initially assessed. The reference section of secondary studies was used to identify additional primary studies, such that only relevant primary studies irrespective of study design were represented in the final list of retained studies. Mobile devices were taken to refer to mobile phones, laptops, personal digital assistants, iPads, and wearable devices, excluding ambulatory health units or desktop computers. Decision tools not integrated into a mobile device, for example, use of paper-based guidelines for decision support were also excluded. All cadres of health workers except social workers, support staff, dentists, pharmacists, and psychiatrists were included. Use of mHealth for patient shared decision-making, self-management, treatment adherence, or patient reminders was an exclusion criterion. In addition, selected interventions had to be used at the point of care, therefore excluding store and forward or remote monitoring techniques such as teleconsultation, teledermatology, and computerized provider order entry systems. We excluded nonclinical forms of decision support such as ethical or policy decision-making and managerial or administrative decision-making. Additional exclusion criteria included articles on the sole use of mHealth as a geographic information or surveillance system, or for managing patient records and data capture without an additional clinical decision support component. Finally, articles that generally discussed mHealth use or training on ICT in health with no focus on a specific intervention, were excluded.

**Figure 1:**
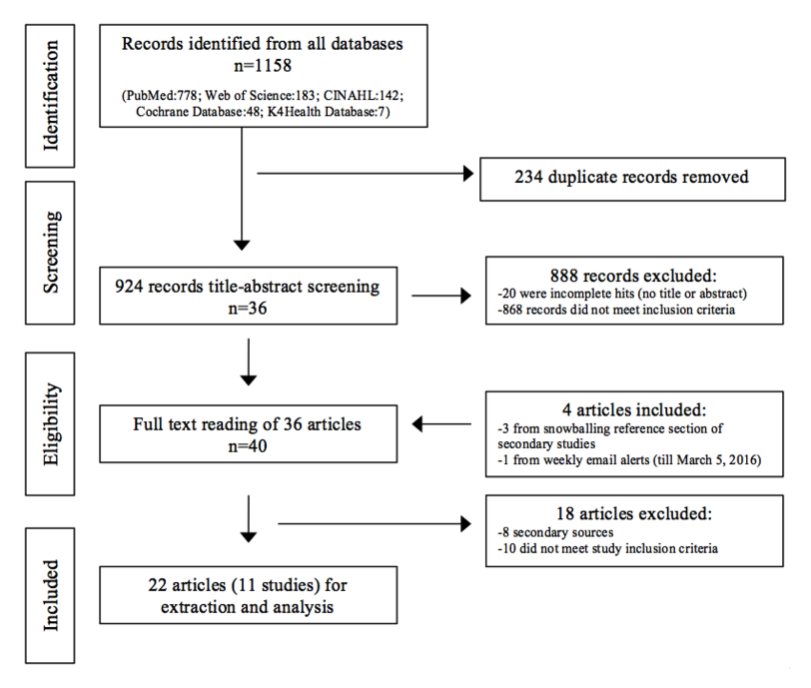
Stepwise flow of study selection.

Supported by multiple discussion meetings, two authors (IOOA and BJAA) applied the inclusion-exclusion criteria on the title and abstract sections of each article, after which 36 articles were selected for full text reading. Where primary reviewers disagreed on inclusion, a third reviewer (MZ) arbitrated. Alongside 3 articles identified from snowballing the reference section of secondary studies and 1 article identified from the weekly alerts, the 36 articles were further assessed for inclusion through full-text reading. Only 22 articles were eventually retained for synthesis and analysis. The stepwise flow is presented in Figure 1.

The subsequent stages of the review (data charting, collating, summarizing, and reporting results) are presented in the following subsections.

### Data Charting

Guided by the review questions, an Excel (version 14.6.6), data charting form was developed and subsequently refined as the papers were read by 2 reviewers. The following information was extracted: general study information including authors, study year, study location, and name of intervention; cadres of health workers targeted; study design and aim; characteristics of the mCDSS; contextual factors of the intervention; expected and reported outcomes of the intervention; descriptive narrative of intervention process and reported facilitators; and barriers to implementation and use of the intervention.

After a clear view of the nature of information in the selected articles was obtained, all articles were exported to MAXQDA version 12.2.0 for coding and data management.

## Results

### Characteristics of Studies

The 22 articles retained in the review represented 11 different studies: m4Change [25], Decision Support and Integrated Record-Keeping (DESIRE) [26], CommCare [27], mPneumonia [28], Basic Antenatal Care Information System (Bacis) [29], TBTech [30], txt2MEDLINE [31], New Algorithm for Managing Childhood Illness Using Mobile Technology (ALMANACH) [32,33], electronic Integrated Management of Childhood Illness (eIMCI) [34-36], Text Messaging of Malaria Guidelines [37-39], and Quality of Maternal and Prenatal Care (QUALMAT) [40-46]). These studies were conducted in 7 sub-Saharan African countries: Kenya, Nigeria, Ghana, Tanzania, Burkina Faso, Botswana, and South Africa. To aid ease of understanding, we took the studies as the unit of analysis and not the different articles in which they are reported. Results are presented in a narrative format.

An overview of each study is presented in Table 1. A detailed profile including study design and outcomes is presented in Multimedia Appendix 3. Key findings are outlined in Textbox 1 at the end.

**Table 1:** Overview of included studies.

Name of intervention	Authors (Year)	Number of articles	Study design	Country	Health domain	Target group	Type of mCDSS^a^
m4Change^b^	McNabb et al (2015) [25]	1	Quantitative pre-post study	Nigeria	Maternal health	Community health (extension) workers	Decision algorithms for antenatal care incorporating clients’ data. Includes audio clips for counseling.
DESIRE (Decision Support and Integrated Record-Keeping)	Vedanthan et al (2015) [26]	1	Qualitative usability and feasibility study	Kenya	Hypertension	Nurses and clinical officers	Electronic records system coupled with algorithm-based decision support with alerts and reminders.
CommCare	Svoronos et al (2010) [27]	1	Qualitative and descriptive	Tanzania	Maternal health	Community health workers	Decision support protocols with reminders and checklists. Incorporates clients’ data for pregnancy monitoring and supervisory oversight.
mPneumonia	Ginsburg et al (2015) [28]	1	Mixed methods usability and feasibility testing	Ghana	Childhood illnesses	Lesser trained health care professionals	Algorithms for managing childhood illnesses integrated with “intelligent” breath counter and pulse oximeter.
Bacis (Basic Antenatal Care Information System)	Horner et al (2013) [29]	1	Before and after cohort study	South Africa	Maternal health	Nurses	Electronic patient information system with protocols to support providers’ action. Includes reminders, alerts, and checklists.
TB Tech	Catalani et al (2014) [30]	1	Mixed methods human-centered design	Kenya	Tuberculosis and HIV	Clinicians	Electronic patient records used to generate individualized reminders and decision support for provider action, education, and behavior change.
txt2MEDLINE	Armstrong et al (2012) [31]	1	Pre-post utility evaluation	Botswana	Different domains	Clinicians of varying cadres	Two-way short messaging service (SMS) of clinical guidelines with MEDLINE query function.
ALMANACH (New Algorithm for Managing Childhood Illness Using Mobile Technology)	Shao et al (2015a, 2015b) [32,33]	2	Controlled noninferiority trial and qualitative study	Tanzania	Childhood illnesses	Clinicians	Diagnostic and treatment algorithm supported by point-of-care tests and simple clinical assessments.
eIMCI (electronic Integrated Management of Childhood Illness)	Mitchell et al (2012, 2013); DeRenzi et al (2008) [34-36]	3	Mixed methods before-after cluster trial	Tanzania	Childhood illnesses	Health care professionals	Electronic protocols for the Integrated Management of Childhood Illnesses (IMCI) for stepwise examination, diagnosis, and management.
Text Messaging of Malaria Guidelines	Jones et al (2012); Zurovac et al (2011, 2012) [37-39]	3	Cluster randomized controlled trial	Kenya	Malaria	Health workers	One-way text messaging on malaria management, supported by unique motivational quotes.
QUALMAT (Quality of Maternal and Prenatal Care)	Blank et al (2013); Dalaba et al (2014, 2015); Mensah et al (2015); Saronga et al (2015); Zakane et al (2014); Duysburgh et al (2016) [40-46]	7	Mixed methods quasi- experimental study	Tanzania; Ghana; Burkina Faso	Maternal and prenatal health	Health professionals (nonphysicians)	Electronic decision support algorithm with data integration. Includes training materials and an electronic partograph.

^a^mCDSS: mobile clinical decision support system.

^b^Although the m4Change study also used the CommCare app, we decided to treat them as independent studies because the interventions were only similar on a technical level and not part of an integrated multicountry study.

### Mobile Clinical Decision Support Systems (mCDSS): Contexts, Purpose, and Features

Alone or as part of a multicountry study, Tanzania had the most number of studies (n=4) on the use of mHealth for clinical decision support, followed by Ghana and Kenya with two studies each. Interventions focused on different domains of health care but were predominantly used in maternal health (n=4), childhood illnesses such as malaria, pneumonia, and diarrhea (n=3), and chronic conditions such as human immunodeficiency virus, tuberculosis, and hypertension (n=2). Lower cadres of health workers (ie, nurses, midwives) or nonclinicians (community health workers) were specifically reported as the target group in the majority of studies. Some studies used broader descriptive terms such as “clinicians” (txt2MEDLINE; TBTech; ALMANACH; DESIRE), “health care professionals” (QUALMAT; mPneumonia), or “health workers” (Text Messaging of Malaria Guidelines), which could also include community health workers and associate clinicians.

Not all studies reported the years of education or clinical experience of the target group. QUALMAT involved associate clinicians having 1-4 years of training, mPneumonia considered users who had up to 2 years of training, and users in the eIMCI had up to 3 years postsecondary school training. Computer literary varied across studies, but most users had no or limited training before the interventions, some of which included training on technology use. Interventions were conducted in either rural (QUALMAT, DESIRE) or mixed (both rural and urban) settings as in the case of TBTech, ALMANACH, and eIMCI. Where reported, most studies were implemented in primary health care facilities (QUALMAT, Bacis, m4Change, eIMCI, mPneumonia).

Studies varied in the type of decision-support; ranging from simple guideline-based two-way (txt2MEDLINE) or one-way (Text Messaging of Malaria Guidelines) text messaging systems to more complex multifunctional systems, which incorporate patients’ data or decision algorithms (m4Change, DESIRE, QUALMAT).

Devices included mobile phones (Text Messaging of Malaria Guidelines, txt2MEDLINE, m4Change, ALMANACH, CommCare), laptops (QUALMAT) and tablets (m4Change, ALMANACH, DESIRE, mPneumonia), or personal digital assistants (eIMCI). With the exception of short messaging service (SMS)–based studies in Kenya (Text Messaging of Malaria Guidelines) and Botswana (txt2MEDLINE) in which users’ personal phones were used, other interventions provided mobile tools and included features for collection and retrieval of patient data.

Three studies incorporated additional components such as tailored motivational quotes (Text Messaging of Malaria Guidelines), performance-based financial and nonfinancial incentives (QUALMAT study), supervisory feedback (CommCare), and diagnostic tools such as pulse oximeters (mPneumonia). Considering infrastructural challenges, some studies also provided batteries, generators, and solar packs (eg, QUALMAT, eIMCI). Local language support was provided in some interventions (QUALMAT, m4Change, eIMCI).

### Mobile Clinical Decision Support Systems (mCDSS): Reported Outcomes

#### Clinical Outcomes

Although the overall aim of a mCDSS should be to improve service delivery, health outcomes, and quality of care, not all papers assessed these. A few studies reported effects of mCDSS on quality of care and showed significant improvement in only a few quality indicators (m4Change, Bacis, QUALMAT). The m4Change project reported statistically significant improvement (*P*<.001) by about four points (from 13.3 to 17.2) in the quality of antenatal care (ANC) services delivered, although not all components of the 25-item quality score were significantly improved. For example, whereas six indicators including client satisfaction improved significantly at endline, a significant decline was recorded for tetanus toxoid coverage, with five other indicators showing no significant improvement. The Bacis study reported an overall increased compliance with using ANC guidelines (from 85.1% to 89.3%), but this was not statistically significant. However, three of nine specific ANC categories (booking patients after week 20, compliance at booking, and use of protocol in patients below 18 years) significantly improved. Similar findings were noted in the larger multicountry QUALMAT study where quality indicators before and after the intervention were mostly not significantly different between intervention and control facilities. Indicators such as history taking, patient monitoring, and total technical performance improved with statistical significance (*P*<.01) postintervention, but remained below maximum satisfactory scores. Unexpectedly, including performance-based incentives (PBI) to enhance health worker motivation in the QUALMAT study did not improve the quality of care.

Overprescription of antibiotics was reduced by about 80% in the ALMANACH study. Although authors suggest this could be the result of improved adherence to evidence-based practice, it was not possible to identify specific factors responsible for this change.

In the SMS intervention of malaria guidelines coupled with motivational messages in Kenya, management of pediatric outpatients improved with statistical significance by 23.7% immediately after the 6-month intervention and was sustained (24.5%) up to 6 months later [39]. Guidelines were found to be most effective for activities that workers previously perceived as unimportant, such as patient counseling, complete physical examination, and follow-up. The authors ascribe this outcome to the perception that guidelines are from an authoritative source, as well as the effectiveness of reminders. Unfortunately, the study did not evaluate the effect of motivational quotes on guidelines adherence.

#### Perceptions of Health Workers

Health workers were generally reported to have positive attitudes toward use of mCDSS, expecting it to make their work easier or simpler, improve efficiency and accuracy, and be more reliable (QUALMAT, TBTech, Text Messaging of Malaria Guidelines). Although not all interventions had this feature, positive attitudes of workers toward mCDSS was linked to their expectation of automatically generated monthly reports, therefore relieving them of this administrative task (CommCare and Basics). mCDSS were additionally perceived to play a supervisory or monitoring role for health workers by ensuring that they followed standard practice. In the one-way SMS guidelines for malaria, the feeling of having an authority figure “looking over ones shoulders” reinforced adherence behavior. The effect of the supervisory feedback component of the CommCare app was not reported.

Studies that included a training module such as QUALMAT were also positively judged. Health workers believed that it met their needs for continuous professional development, therefore increasing competence and self-confidence and resulting in a decreased reliance on peers or referral facilities. Although it did not have a training component, similar perceptions were echoed in the DESIRE study where nurses found the app empowering and perceived it as being able to improve quality of care. The mPneumonia study inferred that in addition to the level of experience of target users, availability of resources such as medical supplies and context of use also influenced disposition of health workers to the mCDSS.

Against the background of long waiting times and understaffed facilities, SMS interventions were appreciated for being easy and concise. The frequency, length, and timing of messages in the Kenyan study on Text Messaging of Malaria Guidelines were important considerations for health workers. Although three out of every four respondents found the frequency of messages (one in the morning and another in the evening, five days a week) adequate, a few considered it excessive and noted the risk of it becoming boring or repetitive.

Health workers raised concerns about increased time periods needed for navigating decision support systems (ALMANACH, QUALMAT). Contrary to initial concerns, workflow assessments in QUALMAT showed that use of the mCDSS did not significantly increase overall time taken to deliver ANC compared with nonintervention sites, although certain tasks such as patient registration and physical examination were found to need twice as much time. This was expected since the standard preintervention paper formats had to be maintained during the intervention. It could also mean that adherence behavior had improved due to the intervention. Studies that measured effects of mCDSS compared with paper systems, such as eIMCI, report that the former was faster and easier to use and improved adherence behavior. The usability assessment of the DESIRE study found that whereas initial use of the system was challenging, given time and frequent use, users found it easier and faster (about 5-20 min) compared with standard paper practice (about 3-30 min) and eventually streamlined it into their workflow. Similar findings were reported in piloting the CommCare app. TBTech was interestingly designed such that both paper and electronic systems were integrated and aligned to existing workflow and organizational processes. This meant that the intervention was not perceived as a big deviation from routine processes of health workers, and therefore easily accepted.

There were reported experiences of conflict and uncertainty when health workers disagreed with recommendations provided by the tool or felt it limited their ability to think for themselves (DESIRE, mPneumonia, TBTech, eIMCI). This unease was especially prominent for workers with insufficient training, in which case their mind-lines (ie, knowledge base) were not reliable. In such situations, health workers were found to rely on patient reports (client-lines). In other studies, the supportive role of mCDSS was emphasized such that health workers realized that they had the authority to override recommendations of algorithms if they believed a different course of action was more appropriate (QUALMAT, Bacis, ALMANACH).

#### Effect of Mobile Clinical Decision Support Systems (mCDSS) on Patient-Provider Relationships

mCDSS was reported to play a role in stimulating or improving trust between patients and providers (ALMANACH, DESIRE, Text Messaging of Malaria Guidelines, eIMCI). For example, patients believed that the mobile tablet was communicating instructions to health workers from more specialized clinicians or from tertiary facilities, which boosted their confidence and trust (eIMCI, DESIRE, ALMANACH).

Compared with paper formats, which patients interpret to indicate lack of knowledge on the part of health workers, mCDSS improved patients’ trust in provider skills, further motivating both parties. An improvement in the technical aspects of care such as physical examination—parts of which may be otherwise skipped—made clients feel attended to and more involved in the care process. Two studies report that use of guidelines or decision algorithms created positive feedback loops whereby more clients were willing to see health workers whose confidence was in turn enhanced (DESIRE, Text Messaging of Malaria Guidelines). A less positive effect was however reported by some nurses who felt that the tablet decreased effectiveness of patient consultation, such as missing nonverbal cues when concentrating on the tablet (DESIRE).

#### Sustainability, Costs, and Cost-Effectiveness of Mobile Clinical Decision Support Systems (mCDSS)

The disposition of health care workers to use mCDSS was not consistent with perceived benefits (ALMANACH, CommCare, txt2MEDLINE). For example, although majority of health workers (79-86%) reported that they would use the txt2MEDLINE system daily or weekly, the initial surge in using the system dropped after a few days. Similar but yet unexplained low levels of use were reported in some study sites under the ALMANACH intervention despite high positive attitudes and enthusiasm for the support system. CommCare suspects that drop in reporting rates after the pilot period was due to technical issues or lack of effective monitoring and supervision. Use of unique motivational messages suggests that such strategies could extend the novelty effect and increase chances of long-term adoption (Text Messaging of Malaria Guidelines).

According to eIMCI study, time efficiency of using the device was an indicator of its sustainability for routine use. Users’ level of literacy and familiarity with technological gadgets were also reported to influence sustained use. For example, the Bacis study found that younger computer literate nurses were more enthusiastic and responsive to the intervention than older nurses.

Only two interventions presented a cost analysis (Text Messaging of Malaria Guidelines, QUALMAT), whereas the Bacis study reported only total cost of study implementation (US $160,000). Over a 6-month period during which 33,361 text messages were sent to 150 phone numbers, US $19,342 was spent in the Text Messaging of Malaria Guidelines study. Most (45%) of this was used to develop and pretest the service with only 13% of costs going toward actual sending of text messages and monitoring of the system. Under study conditions, cost per additional child correctly managed was US $0.5. In the QUALMAT intervention, installation costs varied widely per country—US $186,000 in Tanzania and US $23,000 in Ghana, 77% and 48% of which was spent on the preoperational phases, respectively. These differences were explained by differing contexts, resources, and expenditures needed in each country. Of note is the conclusion that up to US $1060 was required to train a nurse to use the system for a year and about US $21,000 will be required to install and operate mCDSS for 1 year in a similar rural setting.

### Facilitators and Barriers to Mobile Clinical Decision Support Systems (mCDSS) Use

#### Technical and Infrastructural

Poor cellular network coverage and nonfunctional hardware were technical barriers to implementation and use of mCDSS (DESIRE, QUALMAT). Programs such as TBTech built on the work process of existing systems such that decision support functions could be maintained even in situations where electricity or the Internet was unavailable. Some designs allow users input and retrieve data even when offline (m4Change, DESIRE, CommCare, and mPneumonia). One study found that by creating informal communities of practice involving peers with prior experience of mHealth, technical challenges were better managed by program managers (DESIRE).

#### Dual Workload

In the context of low staffing and high caseloads, the concern that mCDSS would further increase workload was a frequently reported barrier to usage (QUALMAT, ALMANACH, DESIRE, mPneumonia). Most ALMANACH users reported that lack of financial incentives demotivated their use of the system although use of financial incentives in the QUALMAT intervention had no additional effect. Equally important is the role that perceived benefit of mCDSS use plays in facilitating its use. Where it was seen as better than current practice (DESIRE), useful for reporting (CommCare), accessing information (txt2MEDLINE), innovative and relevant to their work (Text Messaging of Malaria Guidelines, QUALMAT), health workers were more favorably disposed to its use.

#### Training

Multiple studies found that investing in initial and refresher training was a key facilitator and motivator for effective use of mCDSS (QUALMAT, DESIRE, mPneumonia, Text Messaging of Malaria Guidelines). The need for technical training was higher in older workers (Bacis) with low computer literacy, compared with younger health workers or those who used the system on their personal mobile phones (txt2MEDLINE). Contrarily, another study reported that initial technical difficulties encountered by health workers existed irrespective of sociodemographic and computer literacy levels (ALMANACH).

#### Supervisory Support

The role of technical and supervisory support from both the project team and formal supervisors was thought to be important in keeping users motivated (QUALMAT, CommCare, DESIRE). Delays between training and program implementation could lead to decreased skill, motivation, and general disposition to the intervention. The perception that decision support algorithms are based on updated best practices from a trusted source (national or international body) was also reported as a facilitator of use (Text Messaging of Malaria Guidelines).

#### Ownership

Multistakeholder engagement and ownership needed to be addressed as early as the design phase and before implementation (QUALMAT, Bacis, TBTech). Experiences of the QUALMAT team showed that poor ownership by local stakeholders could lead to suboptimal program outcomes despite including incentives.

#### Health System and Resource Barriers

Health system issues such as unavailability of medicines (m4Change, ALMANACH), health commodities, understaffing (ALMANACH), and the ability to trigger the referral chain when needed, served as facilitating or inhibiting factors to evidence-based practice. Taking these into account, implementation of TBTech included supply chain management, provider training on clinical knowledge, hardware purchase, and maintenance and provision of mobile radiology units. Resource barriers included the need for airtime and financial support to maintain the system (DESIRE, Text2MEDLINE).

**Key findings.**

- mCDSS have been used in a range of 11 interventions in sub-Saharan Africa, with a predominant focus on lower cadre workers, maternal health, and at primary health care level in rural settings.
- With a few exceptions, most interventions were usability or feasibility pilot studies using small sample sizes.
- Although individual service delivery components show improvement, existing evidence does not support the ability of mCDSS to improve quality of care or clinical outcomes in sub-Saharan Africa.
- Use of mCDSS can improve patient-provider relationships through increased trust and confidence in health service delivery.
- mCDSS may create conflicts in clinical decision-making when expert knowledge of health workers conflicts with recommendations of the expert system.
- Although health workers are generally enthusiastic about mCDSS use, there are concerns about its effects on increased workload, altered workflow, and technical challenges, which hinder adoption and sustainability in routine care.
- Facilitators and barriers to use of mCDSS include technical, infrastructural and supervisory support, ownership, and health system constraints.

## Discussion

### Principal Findings

This review synthesized evidence on the use of mobile technology as a clinical decision support system in Africa. Evidence indicates significant support for using mCDSS to improve health worker performance and service delivery specifically within sub-Saharan Africa. However, evidence is insufficient regarding their effects on the quality of care. Key findings are highlighted in Textbox 1.

Weak study designs, short intervention periods, and small sample sizes may explain this gap, although, even from more robust studies, the link to clinical outcomes is largely lacking [47]. Two studies, however, reported statistically significant (m4Change) and even sustained effects (SMS for Malaria Guidelines) on quality of care and provider behavior respectively, which is similar to reports on the ability of mCDSS to improve adherence to guidelines, evidence-based practice, and patient outcomes [48,49]. Other reviews have reported studies showing effects on guideline adherence or patient outcomes, which were either not statistically significant or suboptimal [47]. Specific features of computerized decision aids could enhance (eg, content control) or constrain (eg, patient narratives) the quality of decision-making [11], but we could not establish direct links between study outcomes and features of the mCDSS used. Significant improvement in clinical practice has been shown in decision support systems focused on clinicians and associate clinicians (physician assistants and nurses) [48]; however, none of the interventions compared perceptions and outcomes across different health worker cadres.

Unsustained enthusiasm regarding mCDSS use reflects the novelty effect, which in addition to perceived risk or reward can influence technological adoption [50]. High expectations or inaccurate perceptions of the capability of mobile devices may explain why some workers used the system more than others, as was the case in the ALMANACH study. It could also be due to short training or intervention periods, limiting ability of users to become familiar with the system, and to modify their expectations. According to Rogers’ theory on the diffusion of innovations [51], individual, systemic, and innovation-related factors influence the adoption of innovations and their potential to effectively influence systemic change. Perceived usefulness of mCDSS in light of users’ perception on its effect on their workload, alongside other institutional and resource barriers, could have hindered the transition from early to sustained adoption by health care workers. Although the relatively short duration characterizing many mHealth pilots hinder the ability to evaluate rate and effect of adoption over time, a human-centered, multistakeholder approach to design and implement these technologies has been suggested as a way to mitigate resistance and encourage efficient integration into complex environments such as health systems [27,30,40-46]. Although some of the studies in this review used strategies such as training, supervision, and financial incentives to motivate the adoption and utilization of mCDSS, there were mixed reports about their effectiveness. Direct or indirect supervisory support may additionally trigger the Hawthorne effect, influencing mCDSS adoption. Despite health worker concerns, evidence showed that consultation time was not significantly increased due to these innovations. Future studies need to understand how mCDSS influences workflow patterns—the goal of which is to improve time efficiency while retaining quality services, and they should aim to identify how mHealth innovations can be designed and implemented to effectively become an integral part of the systems in which they are introduced.

In a study on factors that influence decision-making of frontline health workers in Ghana, health workers’ tacit knowledge (mind-lines) was the default mode for clinical decision-making, with guidelines used only when they were easily accessible and simple to use [52]. The risk of overreliance on the recommendations of mCDSS (e-lines) above provider knowledge and experience, and the conflict that could result has been established in the discussions on limitations of decision support systems [49]. However, there is equal need to consider that mind-lines of health workers may be inaccurate and shaped by flawed perceptions, insufficient clinical training, and sociocultural norms [53]. The flexibility to override decision support recommendations may therefore need to be balanced with system accuracy and training or experience of users.

Findings that providers were more engaged in the care process during mCDSS use contradict anecdotal perceptions that interpersonal relationships are decreased with the use of electronic devices. Although inconclusive, whereas these effects on improved patient-provider relationships could be due to improved adherence to standard evidence-based practice, they could also be purely psychological and inflated. Future before-after studies that assess attitudinal and interpersonal changes are therefore needed.

There were no additional studies reporting the use of PBI on implementation and use of mCDSS in sub-Saharan Africa. A US study which included financial incentives (US $500-800) to nurses and clinicians over a 6-month period reported that use of the intervention was moderately sustained even after the incentives were stopped [54]. Other studies in high-resource contexts have highlighted the beneficial role of incentives at a facility level [55]. In one country site of the QUALMAT study, financial (€4297) and nonfinancial (trophies, a camera, a cell phone, and acknowledgment letters) incentives were provided at facility and individual levels respectively (p.34) [56]. Although these may have stimulated use of mCDSS, quality of care did not improve. Further investigation is needed regarding the benefits of financial or nonfinancial incentives in implementing and sustaining mCDSS use, and at what level PBI are most effective. This also highlights the multiplicity of factors that need to be taken into account to achieve effective clinical decision-making support interventions.

### Recommendations for Policy, Practice and Research

A major concern of policy makers regarding added benefits of adopting mHealth is related to its cost and cost-effectiveness. Although only 2 of the 11 studies reported cost implications, willingness of stakeholders to share costs is important for continuity and sustainability. Studies that made use of personal phones of health workers (Text Messaging of Malaria Guidelines, txt2MEDLINE) utilized an indirect form of cost sharing. Assessment of stakeholders’ willingness-to-pay or cost-sharing models could prompt consideration for scaling-up successful pilot interventions. Evidence points to low-cost implications and higher acceptability with SMS-based mCDSS. A Chinese study found that compared with standard paper formats, text messages were about 280 times cheaper for stimulating guideline use [57]. Although there was agreement on the ease of its use, most respondents found that the messages, which were received once daily three times a week, were too short and infrequent. It is crucial to conduct additional studies that show how and when timing, frequency, and length of text message mCDSS interventions are most suitable. Regular updates of decision support software could also minimize the risk of information being perceived as redundant.

Clinical decision-making is only one aspect of the continuum of care. Success of using electronic support as a “magic” tool is hampered by other deficiencies in the health system such as not being able to act on recommendations [33]. This may possibly explain suboptimal effects on quality of service delivery. The extent to which mCDSS increases competencies of lower cadre health workers needs to be investigated so that task-shifting strategies can better leverage technological innovation. Rigorous evaluation methodology could shed more light on outcome and impact of the use of mHealth for clinical decision support especially taking into consideration different contexts, various cadres of health workers, and their levels of experience and training. As health care systems are increasingly incorporating technological and ICT-based interventions into routine practice, training of all health professionals should be adapted to include this competence.

### Limitations

Although the evidence in this review spans interventions executed within the last ten years, resources did not allow us to engage in translations of articles in other languages, which implies that we may have excluded some relevant articles from French-speaking countries. Additionally, although we recognized that we may have gained more insight into the different interventions if we had included a consultation stage in the review process [20], due to time constraints, we did not contact study authors for additional information or further ongoing research. In contrast to systematic reviews, the absence of quality assessment of papers included in scoping reviews makes findings hard to generalize and the effectiveness of studies difficult to weigh [20]. Despite these limitations, we believe that the breadth and depth of evidence presented here is sufficiently relevant for the aims of this review.

### Conclusions

The volume of evidence presented on the use of mobile technology as a clinical decision support system in sub-Saharan Africa is an indication of growth in the domain and its potential for improving health service delivery in low-resource settings. Several evidence gaps need to be addressed, including specific mechanisms underlying use, sustainability, and effects of mCDSS on quality of care and their ability to be fully integrated into routine practice. In light of the effect that differences in health worker cadre, training, and intervention context could have on utilization and outcomes of mCDSS, future research should adopt comparative analyses in order to identify for whom these programs work best. It is also needful to understand in what contexts, why, how, and at what costs, mCDSS lead to changes in health worker performance. Although quality of service delivered by these interventions on a clinical and individual level is yet to be fully explored, the evidence gathered is useful for informing future policy, practice, and research.

## Appendix

Multimedia Appendix 1 Search strategy.

Multimedia Appendix 2 Search strategy for different databases.

Multimedia Appendix 3 Detailed profile of included studies.

## Abbreviations

ANC: antenatal care
Bacis: Basic Antenatal Care Information System
DESIRE: Decision Support and Integrated Record-Keeping
eIMCI: electronic Integrated Management of Childhood Illness
ICT: information and communications technology
mCDSS: mobile clinical decision support system
mHealth: mobile health
PBI: performance-based incentives
SMS: short messaging service
QUALMAT: Quality of Maternal and Prenatal Care
