# Supplementary material for: mHealth for Clinical Decision-Making in Sub-Saharan Africa: A Scoping Review
Source: JMIR Mhealth Uhealth. 2017 Mar 23;5(3):e38. doi: 10.2196/mhealth.7185 (PMC5383806; doi:10.2196/mhealth.7185)
Supplement: Multimedia Appendix 1 [file mhealth_v5i3e38_app1.pdf]

Concept	Syntax
mobile health	Telemedicine[mh] OR telenursing[mh] OR User-Computer Interface[mh] OR cell phones[mh] OR public health informatics[mh] OR medical informatics[mh] OR nursing informatics[mh] OR computers, handheld[mh] OR internet[mh] OR Mobile Applications[mh] OR mobile health*[tiab] OR mHealth*[tiab] OR m-health*[tiab] OR ehealth*[tiab] OR e-health*[tiab] OR mobile health[tiab] OR digital health[tiab] OR app[tiab] OR apps[tiab] OR smartphone*[tiab] OR phone application[tiab] OR phone applications[tiab] OR cellphone application[tiab] OR cellphone applications[tiab] OR telephone application[tiab] OR telephone applications[tiab] OR mobile application[tiab] OR mobile applications[tiab] OR mobile technolog*[tiab] OR health technolog*[tiab] OR health application[tiab] OR health applications[tiab] OR internet[tiab] OR iPad[tiab] OR sms[tiab] OR text messag*[tiab] OR USSD[tiab] OR pda[tiab] OR laptop*[tiab] OR palmtop*[tiab] OR palm-top*[tiab] OR Personal Digital Assistant*[tiab] OR computer*[tiab] OR cell phone*[tiab] OR cellular phone*[tiab] OR smart phone*[tiab]
Decision-making<br>OR<br>Quality of care	Decision-making[mh] OR decision-making, computer-assisted[mh] OR evidence-based medicine[mh] OR evidence-based nursing[mh] OR decision support techniques[mh] OR decision support systems, clinical[mh] OR guideline adherence[mh] OR health care quality, access, and evaluation[mh] OR quality of health care[mh] OR workflow[mh] OR patient care[mh] OR delivery of health care[mh] OR health services[mh] OR patient care management[mh] OR decision-making[tiab] OR decision support[tiab] OR Evidence-based[tiab] OR decision aid*[tiab] OR guideline*[tiab] OR decision process*[tiab] OR decision tool*[tiab] OR health service*[tiab] OR health care quality[tiab] OR healthcare quality[tiab] OR health outcome*[tiab] OR quality of health[tiab] OR quality of care[tiab] OR quality care[tiab] OR competen*[tiab] OR best practic*[tiab] OR patient care[tiab]
Health care workers	Health personnel[mh] OR nurse[tiab] OR nurses[tiab] OR physician[tiab] OR physicians[tiab] OR health provider[tiab] OR health providers[tiab] OR health care provider[tiab] OR health care providers[tiab] OR healthcare provider[tiab] OR healthcare providers[tiab] OR health worker[tiab] OR health workers[tiab] OR midwife[tiab] OR midwives[tiab] OR health care worker[tiab] OR health care workers[tiab] OR healthcare worker[tiab] OR healthcare workers[tiab] OR community health worker[tiab] OR community health workers[tiab] OR practitioner[tiab] OR practitioners[tiab] OR clinician[tiab] OR clinicians[tiab] OR doctor[tiab] OR doctors[tiab] OR clinical officer[tiab]

	OR clinical officers[tiab] OR medical personnel[tiab] OR health professional[tiab] OR health professionals[tiab] OR frontline provider[tiab] OR frontline providers[tiab] OR frontline worker[tiab] OR frontline workers[tiab] OR traditional birth attend*[tiab] OR front line provider*[tiab] OR front line worker*[tiab]
Africa	Africa[mh] OR africa[tiab] OR Cameroon[tiab] OR Central African Republic[tiab] OR Chad[tiab] OR Congo[tiab] OR Democratic Republic of the Congo[tiab] OR Equatorial Guinea[tiab] OR Gabon[tiab] OR Burundi[tiab] OR Djibouti[tiab] OR Eritrea[tiab] OR Ethiopia[tiab] OR Kenya[tiab] OR Rwanda[tiab] OR Somalia[tiab] OR Sudan[tiab] OR Tanzania[tiab] OR Burundi[tiab] OR Djibouti[tiab] OR Uganda[tiab] OR Angola[tiab] OR Botswana[tiab] OR Lesotho[tiab] OR Malawi[tiab] OR Mozambique[tiab] OR Namibia[tiab] OR South Africa[tiab] OR Swaziland[tiab] OR Zambia[tiab] OR Zimbabwe[tiab] OR Benin[tiab] OR Burkina Faso[tiab] OR Cape Verde[tiab] OR Ivory Coast[tiab] OR Cote d'Ivoire[tiab] OR Gambia[tiab] OR Ghana[tiab] OR Guinea[tiab] OR Guinea-Bissau[tiab] OR Liberia[tiab] OR Mali[tiab] OR Mauritania[tiab] OR Niger[tiab] OR Nigeria[tiab] OR Senegal[tiab] OR Sierra Leone[tiab] OR Togo[tiab] OR Algeria[tiab] OR Egypt[tiab] OR Libya[tiab] OR Morocco[tiab] OR Tunisia[tiab]
