# Supplementary material for: mHealth for Clinical Decision-Making in Sub-Saharan Africa: A Scoping Review
Source: JMIR Mhealth Uhealth. 2017 Mar 23;5(3):e38. doi: 10.2196/mhealth.7185 (PMC5383806; doi:10.2196/mhealth.7185)
Supplement: Multimedia Appendix 2 [file mhealth_v5i3e38_app2.pdf]

PubMed (25th December 2015)

Search	Query
#6	Search #1 AND #2 AND #3 AND #4 Sort by: Relevance Filters: English
#5	Search #1 AND #2 AND #3 AND #4
#4	Search (Africa[mh] OR africa[tiab] OR Cameroon[tiab] OR Central African Republic[tiab] OR Chad[tiab] OR Congo[tiab] OR Democratic Republic of the Congo[tiab] OR Equatorial Guinea[tiab] OR Gabon[tiab] OR Burundi[tiab] OR Djibouti[tiab] OR Eritrea[tiab] OR Ethiopia[tiab] OR Kenya[tiab] OR Rwanda[tiab] OR Somalia[tiab] OR Sudan[tiab] OR Tanzania[tiab] OR Burundi[tiab] OR Djibouti[tiab] OR Uganda[tiab] OR Angola[tiab] OR Botswana[tiab] OR Lesotho[tiab] OR Malawi[tiab] OR Mozambique[tiab] OR Namibia[tiab] OR South Africa[tiab] OR Swaziland[tiab] OR Zambia[tiab] OR Zimbabwe[tiab] OR Benin[tiab] OR Burkina Faso[tiab] OR Cape Verde[tiab] OR Ivory Coast[tiab] OR Cote d'Ivoire[tiab] OR Gambia[tiab] OR Ghana[tiab] OR Guinea[tiab] OR Guinea-Bissau[tiab] OR Liberia[tiab] OR Mali[tiab] OR Mauritania[tiab] OR Niger[tiab] OR Nigeria[tiab] OR Senegal[tiab] OR Sierra Leone[tiab] OR Togo[tiab] OR Algeria[tiab] OR Egypt[tiab] OR Libya[tiab] OR Morocco[tiab] OR Tunisia[tiab])
#3	Search (Health personnel[mh] OR nurse[tiab] OR nurses[tiab] OR physician[tiab] OR physicians[tiab] OR health provider[tiab] OR health providers[tiab] OR health care provider[tiab] OR health care providers[tiab] OR healthcare provider[tiab] OR healthcare providers[tiab] OR health worker[tiab] OR health workers[tiab] OR midwife[tiab] OR midwives[tiab] OR health care worker[tiab] OR health care workers[tiab] OR healthcare worker[tiab] OR healthcare workers[tiab] OR community health worker[tiab] OR community health workers[tiab] OR practitioner[tiab] OR practitioners[tiab] OR clinician[tiab] OR clinicians[tiab] OR doctor[tiab] OR doctors[tiab] OR clinical officer[tiab] OR clinical officers[tiab] OR medical personnel[tiab] OR health professional[tiab] OR health professionals[tiab] OR frontline provider[tiab] OR frontline providers[tiab] OR frontline worker[tiab] OR frontline workers[tiab] OR traditional birth attend*[tiab] OR front line provider*[tiab] OR front line worker*[tiab])
#2	Search (Decision Making[mh] OR decision making, computer-assisted[mh] OR evidence-based medicine[mh] OR evidence-based nursing[mh] OR decision support techniques[mh] OR decision support systems, clinical[mh] OR guideline adherence[mh] OR health care quality, access, and evaluation[mh] OR quality of health care[mh] OR workflow[mh] OR patient care[mh] OR delivery of health care[mh] OR health services[mh] OR patient care management[mh] OR decision making[tiab] OR decision support[tiab] OR Evidence-based[tiab] OR decision aid*[tiab] OR guideline*[tiab] OR decision process*[tiab] OR decision tool*[tiab] OR health service*[tiab] OR health care quality[tiab] OR healthcare quality[tiab] OR health outcome*[tiab] OR quality of health[tiab] OR quality of care[tiab] OR quality

	care[tiab] OR competen*[tiab] OR best practic*[tiab] OR patient care[tiab])
#1	Search (Telemedicine[mh] OR telenursing[mh] OR User-Computer Interface[mh] OR cell phones[mh] OR public health informatics[mh] OR medical informatics[mh] OR nursing informatics[mh] OR computers, handheld[mh] OR internet[mh] OR Mobile Applications[mh] OR mobile health*[tiab] OR mhealth*[tiab] OR m-health*[tiab] OR ehealth*[tiab] OR e-health*[tiab] OR mobile health[tiab] OR digital health[tiab] OR app[tiab] OR apps[tiab] OR smartphone*[tiab] OR phone application[tiab] OR phone applications[tiab] OR cellphone application[tiab] OR cellphone applications[tiab] OR telephone application[tiab] OR telephone applications[tiab] OR mobile application[tiab] OR mobile applications[tiab] OR mobile technolog*[tiab] OR health technolog*[tiab] OR health application[tiab] OR health applications[tiab] OR internet[tiab] OR iPad[tiab] OR sms[tiab] OR text messag*[tiab] OR USSD[tiab] OR pda[tiab] OR laptop*[tiab] OR palmtop*[tiab] OR palm-top*[tiab] OR Personal Digital Assistant*[tiab] OR computer*[tiab] OR cell phone*[tiab] OR cellular phone*[tiab] OR smart phone*[tiab])

Cochrane (24th December 2015)

Search	Query
#1	MeSH descriptor: [Telemedicine] explode all tress
#2	MeSH descriptor: [Telenursing] explode all trees
#3	MeSH descriptor: [User-Computer Interface] explode all tress
#4	MeSH descriptor: [Cell Phones] explode all tress
#5	MeSH descriptor: [Public Health Informatics] explode all tress
#6	MeSH descriptor: [Medical Informatics] explode all tress
#7	MeSH descriptor: [Nursing Informatics] explode all tress
#7	MeSH descriptor: [Computers, Handheld] explode all tress
#9	MeSH descriptor: [Internet] explode all tress
#10	MeSH descriptor: [Mobile Applications] explode all tress
#11	#1 or #2 or #3 or #4 or #5 or #6 or #7 or #8 or #9 or #10
#12	(#11) or ("mobile health*" or mhealth* or "m-health*" or ehealth* or "e-health*" or "digital health" or app or apps or smartphone* or "phone application" or "phone applications" or "cellphone application" or "cellphone applications" or "telephone application" or "telephone applications" or "mobile application" or "mobile

	applications" or "mobile technolog*" or "health technolog*" or "health application" or "health applications" or internet or iPad or sms or "text messag*" or USSD or pda or laptop* or palmtop* or "palm-top*" or "Personal Digital Assistant*" or computer* or "cell phone*" or "cellular phone*" or "smart phone*"):ab,ti,kw
#13	MeSH descriptor: [Africa] explode all tress
#14	(#13) or (Africa or Cameroon or "Central African Republic" or Chad or Congo or "Equatorial Guinea" or Gabon or Burundi or Djibouti or Eritrea or Ethiopia or Kenya or Rwanda or Somalia or Sudan or Tanzania or Burundi or Djibouti or Uganda or Angola or Botswana or Lesotho or Malawi or Mozambique or Namibia or "South Africa" or Swaziland or Zambia or Zimbabwe or Benin or "Burkina Faso" or "Cape Verde" or "Ivory Coast" or "Cote d'Ivoire" or Gambia or Ghana or Guinea or "Guinea-Bissau" or Liberia or Mali or Mauritania or Niger or Nigeria or Senegal or "Sierra Leone" or Togo or Algeria or Egypt or Libya or Morocco or Tunisia):ab,ti,kw
#15	MeSH descriptor: [Health Personnel] explode all trees
#16	(#15) or (nurse or nurses or physician or physicians or "health provider" or "health providers" or "health care provider" or "health care providers" or "healthcare provider" or "healthcare providers" or "health worker" or "health workers" or midwife or midwives or "health care worker" or "health care workers" or "healthcare worker" or "healthcare workers" or "community health worker" or "community health workers" or practitioner or practitioners or clinician or clinicians or doctor or doctors or "clinical officer" or "clinical officers" or "medical personnel" or "health professional" or "health professionals" or "frontline provider" or "frontline providers" or "frontline worker" or "frontline workers" or "traditional birth attend*" or "front line provider*" or "front line worker*"):ab,ti,kw
#17	MeSH descriptor: [Decision Making] explode all trees
#18	MeSH descriptor: [Evidence-Based Medicine] explode all trees
#19	MeSH descriptor: [Decision Making, Computer-Assisted] explode all trees
#20	MeSH descriptor: [Evidence-Based Nursing] explode all trees
#21	MeSH descriptor: [Decision Support Techniques] explode all trees
#22	MeSH descriptor: [Decision Support Systems, Clinical] explode all trees
#23	MeSH descriptor: [Guideline Adherence] explode all trees
#24	MeSH descriptor: [Health Care Quality, Access, and Evaluation] explode all trees
#25	MeSH descriptor: [Workflow] explode all trees
#26	MeSH descriptor: [Patient Care] explode all trees
#27	MeSH descriptor: [Health Services] explode all trees

#28	MeSH descriptor: [Patient Care Management] explode all trees
#29	#17 or #18 or #19 or #20 or #21 or #22 or #23 or #24 or #25 or #26 or #27 or #28
#30	(#29) or ("decision making" or "decision support" or "Evidence-based" or "decision aid*" or guideline* or "decision process*" or "decision tool*" or "health service*" or "health care quality" or "healthcare quality" or "health outcome*" or "quality of health" or "quality of care" or "quality care" or competen* or "best practic*" or "patient care"):ab,ti,kw
#31	#12 and #30 and #16 and #14

Web of Science Core Collection (24th December 2015)

Search	Query
# 6	(#1 AND #2 AND #3 AND #4) *AND* LANGUAGE: (English)<br>*Indexes=SCI-EXPANDED, SSCI, A&HCI, ESCI Timespan=All years*
# 5	#1 AND #2 AND #3 AND #4<br>*Indexes=SCI-EXPANDED, SSCI, A&HCI, ESCI Timespan=All years*
# 4	TS=(Africa or Cameroon or "Central African Republic" or Chad or Congo or "Equatorial Guinea" or Gabon or Burundi or Djibouti or Eritrea or Ethiopia or Kenya or Rwanda or Somalia or Sudan or Tanzania or Burundi or Djibouti or Uganda or Angola or Botswana or Lesotho or Malawi or Mozambique or Namibia or "South Africa" or Swaziland or Zambia or Zimbabwe or Benin or "Burkina Faso" or "Cape Verde" or "Ivory Coast" or "Cote d'Ivoire" or Gambia or Ghana or Guinea or "Guinea-Bissau" or Liberia or Mali or Mauritania or Niger or Nigeria or Senegal or "Sierra Leone" or Togo or Algeria or Egypt or Libya or Morocco or Tunisia)<br>*Indexes=SCI-EXPANDED, SSCI, A&HCI, ESCI Timespan=All years*
# 3	TS=(nurse or nurses or physician or physicians or "health provider" or "health providers" or "health care provider" or "health care providers" or "healthcare provider" or "healthcare providers" or "health worker" or "health workers" or midwife or midwives or "health care worker" or "health care workers" or "healthcare worker" or "healthcare workers" or "community health worker" or "community health workers" or practitioner or practitioners or clinician or clinicians or doctor or doctors or "clinical officer" or "clinical officers" or "medical personnel" or "health professional" or "health professionals" or "frontline provider" or "frontline providers" or "frontline worker" or "frontline workers" or "traditional birth attend*" or "front line provider*" or "front line worker*")<br>*Indexes=SCI-EXPANDED, SSCI, A&HCI, ESCI Timespan=All years*

# 2	TS=(decision making or decision support or Evidence based or "decision aid*" or guideline* or "decision process*" or "decision tool*" or "health service*" or "health care quality" or "healthcare quality" or "health outcome*" or "quality of health" or "quality of care" or "quality care" or competen* or "best practic*" or "patient care")<br><br>*Indexes=SCI-EXPANDED, SSCI, A&HCI, ESCI Timespan=All years*
# 1	TS=("mobile health*" or mhealth* or "m-health*" or ehealth* or "e-health*" or "digital health" or app or apps or smartphone* or "phone application" or "phone applications" or "cellphone application" or "cellphone applications" or "telephone application" or "telephone applications" or "mobile application" or "mobile applications" or "mobile technolog*" or "health technolog*" or "health application" or "health applications" or internet or iPad or sms or "text messag*" or USSD or pda or laptop* or palmtop* or "palm-top*" or "Personal Digital Assistant*" or computer* or "cell phone*" or "cellular phone*" or "smart phone*")<br><br>*Indexes=SCI-EXPANDED, SSCI, A&HCI, ESCI Timespan=All years*

CINAHL (24th December 2015)

Search	Query
S5	S1 AND S2 AND S3 AND S4 (English Language)
S4	(MH "Africa+") OR TI (Africa or Cameroon or "Central African Republic" or Chad or Congo or "Equatorial Guinea" or Gabon or Burundi or Djibouti or Eritrea or Ethiopia or Kenya or Rwanda or Somalia or Sudan or Tanzania or Burundi or Djibouti or Uganda or Angola or Botswana or Lesotho or Malawi or Mozambique or Namibia or "South Africa" or Swaziland or Zambia or Zimbabwe or Benin or "Burkina Faso" or "Cape Verde" or "Ivory Coast" or "Cote d'Ivoire" or Gambia or Ghana or Guinea or "Guinea-Bissau" or Liberia or Mali or Mauritania or Niger or Nigeria or Senegal or "Sierra Leone" or Togo or Algeria or Egypt or Libya or Morocco or Tunisia) OR AB (Africa or Cameroon or "Central African Republic" or Chad or Congo or "Equatorial Guinea" or Gabon or Burundi or Djibouti or Eritrea or Ethiopia or Kenya or Rwanda or Somalia or Sudan or Tanzania or Burundi or Djibouti or Uganda or Angola or Botswana or Lesotho or Malawi or Mozambique or Namibia or "South Africa" or Swaziland or Zambia or Zimbabwe or Benin or "Burkina Faso" or "Cape Verde" or "Ivory Coast" or "Cote d'Ivoire" or Gambia or Ghana or Guinea or "Guinea-Bissau" or Liberia or Mali or Mauritania or Niger or Nigeria or Senegal or "Sierra Leone" or Togo or Algeria or Egypt or Libya or Morocco or Tunisia)
S3	(MH "Health Personnel+") OR TI (nurse or nurses or physician or physicians or "health provider" or "health providers" or "health care provider" or "health care providers" or "healthcare provider" or "healthcare providers" or "health worker" or

	"health workers" or midwife or midwives or "health care worker" or "health care workers" or "healthcare worker" or "healthcare workers" or "community health worker" or "community health workers" or practitioner or practitioners or clinician or clinicians or doctor or doctors or "clinical officer" or "clinical officers" or "medical personnel" or "health professional" or "health professionals" or "frontline provider" or "frontline providers" or "frontline worker" or "frontline workers" or "traditional birth attend*" or "front line provider*" or "front line worker*") OR AB (nurse or nurses or physician or physicians or "health provider" or "health providers" or "health care provider" or "health care providers" or "healthcare provider" or "healthcare providers" or "health worker" or "health workers" or midwife or midwives or "health care worker" or "health care workers" or "healthcare worker" or "healthcare workers" or "community health worker" or "community health workers" or practitioner or practitioners or clinician or clinicians or doctor or doctors or "clinical officer" or "clinical officers" or "medical personnel" or "health professional" or "health professionals" or "frontline provider" or "frontline providers" or "frontline worker" or "frontline workers" or "traditional birth attend*" or "front line provider*" or "front line worker*")
S2	(MH "Decision Making+") OR (MH "Professional Practice, Evidence-Based+") OR (MH "Decision Support Techniques+") OR (MH "Decision Support Systems, Clinical") OR (MH "Guideline Adherence") OR (MH "Quality of Health Care+") OR (MH "Patient Care+") OR (MH "Health Care Delivery+") OR (MH "Health Services+") OR (MH "Patient Care Plans+") OR TI ("decision making" OR "decision support" OR "Evidence-based" OR "decision aid*" OR guideline* OR "decision process*" OR "decision tool*" OR "health service*" OR "health care quality" OR "healthcare quality" OR "health outcome*" OR "quality of health" OR "quality of care" OR "quality care" OR competen* OR "best practic*" OR "patient care") OR AB ("decision making" OR "decision support" OR "Evidence-based" OR "decision aid*" OR guideline* OR "decision process*" OR "decision tool*" OR "health service*" OR "health care quality" OR "healthcare quality" OR "health outcome*" OR "quality of health" OR "quality of care" OR "quality care" OR competen* OR "best practic*" OR "patient care")
S1	(MH "Telemedicine+") OR (MH "User-Computer Interface+") OR (MH "Cellular Phone+") OR (MH "Health Informatics+") OR (MH "Computers, Hand-Held+") OR (MH "Internet+") OR (MH "Mobile Applications") OR TI ("mobile health*" OR mhealth* OR "m-health*" OR ehealth* OR "e-health*" OR "digital health" OR app OR apps OR smartphone* OR "phone application" OR "phone applications" OR "cellphone application" OR "cellphone applications" OR "telephone application" OR "telephone applications" OR "mobile application" OR "mobile applications" OR "mobile technolog*" OR "health technolog*" OR "health application" OR "health applications" OR internet OR iPad OR sms OR "text messag*" OR USSD OR pda OR laptop* OR palmtop* OR "palm-top*" OR "Personal Digital Assistant*" OR computer* OR "cell phone*" OR "cellular phone*" OR "smart phone*") OR AB ("mobile health*" OR mhealth* OR "m-

	health*" OR ehealth* OR "e-health*" OR "digital health" OR app OR apps OR smartphone* OR "phone application" OR "phone applications" OR "cellphone application" OR "cellphone applications" OR "telephone application" OR "telephone applications" OR "mobile application" OR "mobile applications" OR "mobile technolog*" OR "health technolog*" OR "health application" OR "health applications" OR internet OR iPad OR sms OR "text messag*" OR USSD OR pda OR laptop* OR palmtop* OR "palm-top*" OR "Personal Digital Assistant*" OR computer* OR "cell phone*" OR "cellular phone*" OR "smart phone*")

mHealth Evidence Knowledge 4 Health Database (24th December 2015**)**

Search String: “decision support” followed by manually combining “decision support" and Tunisia (or Africa or Botswana i.e. country name or continent name) individually for all 60 possibilities.
