# Supplementary material for: mHealth for Clinical Decision-Making in Sub-Saharan Africa: A Scoping Review
Source: JMIR Mhealth Uhealth. 2017 Mar 23;5(3):e38. doi: 10.2196/mhealth.7185 (PMC5383806; doi:10.2196/mhealth.7185)
Supplement: Multimedia Appendix 3 [file mhealth_v5i3e38_app3.pdf]

Name of Intervention<br><br>Authors (Year)	Study design	Country<br><br>Health Domain<br><br>Target Group	Type and Characteristics of mCDSS[a]	Expected Outcome	Reported Outcomes
m4Change[b]<br><br>McNabb et al (2015) [25]	Quantitative pre-post study	Nigeria<br><br>Maternal Health<br><br>CHEW/HCWs	-Mobile phone and Tablet<br>-Guided decision support with algorithms for ANC and client data<br>-Health education audio clips for client counselling<br>-Local language support<br>-Offline functionality<br>-Password protected	-Effect on quality of ANC services<br>-Effect on client satisfaction	-Generally, quality score improved significantly by about 4 points (from 13.3 at baseline to 17.2 at endline)<br>-Not all specific elements of the score significantly improved<br>-Client satisfaction with ANC services significantly improved at end line.
DESIRE (Decision Support and Integrated Record-keeping)<br><br>Vedanthan et al (2015) [26]	Qualitative usability and feasibility study	Kenya<br><br>Hypertension<br><br>Nurses and Clinical Officers	-Tablet-based patient-specific decision-support tool with branching logic algorithm, alerts and reminders.<br>-Integrated with patient data and linked to a central medical records<br>-Offline functionality<br>-Data security via user-authentication, automatic timeouts, encryption and secure transmission system	-Identification of barriers and facilitators to implementing the DESIRE tool	-Technical and human barriers to implementation were identified.<br>-Feasibility themes included facilitators to implementation, provider or patient issues and additional feature requests<br>-Twenty-one unique critical incidents identified in usability testing.

CommCare<br><br>Svoronos et al (2010) [27]	Qualitative and descriptive	Tanzania<br><br>Maternal Health<br><br>CHWs	-Phone-based CommCare application including user-controlled guided decision support tool, checklist and follow-up reminders for quality improvement<br>-Use of patient data for referral support and monitoring<br>-Supervisory feedback and report generation<br>-Offline functionality<br>-Password protected	-Develop CommCare module	-Application was well received by CHWs and found to improve and standardize service delivery (identification, follow up and referral)<br>-Ease and comfort of use reported after some training<br>-Use of tool declined postimplementation
mPneumonia Ginsburg et al (2015) [28]	Mixed methods usability and feasibility testing	Ghana<br><br>Childhood Illnesses<br><br>Lesser trained health care professionals	-Tablet-based tool with IMCI algorithm and decision-making protocol<br>-Coupled with “intelligent” electronic breath counter and paediatric pulse oximeter with visual, auditory, and vibratory feedback<br>-Offline functionality<br>-English language support<br>-Password protected	-Design and development of mPneumonia to improve diagnostic accuracy and facilitate guideline adherence by HCWs.	-HCWs positively responded to the mCDSS and found it innovative and easy.<br>-HCWs preferred it to standard (paper) practice, anticipating accurate and easier care management<br>-Concerns about maintenance and theft<br>-Identified 17 critical and 9 noncritical usability issues<br>-Increased ease of use with repetition<br>-Suggested at least 2 days (16.3hours) of training

Bacis (Basic Antenatal Care Information System)<br><br>Horner et al (2013) [29]	Before and after cohort study	South Africa<br><br>Maternal Health<br><br>Nurses	-Electronic patient information system with clinical decision support for maternal health via protocols, checklists, and a rule and knowledge base with alerts and reminders<br>-Supports patient data entry for classification, follow-up and referral, with validation and completeness checks	-Usability and acceptability review<br>-Compliance (i.e. completion and response) performance for maternity care protocols and the antenatal care checklist	-Improved compliance from 85% to 89% although not statistically significant<br>-Out of nine specific categories for measuring compliance, three (compliance at booking, patients <18 years & booking patients after week 20) showed statistically significant results compared to standard practice using paper<br>-Better suited for younger computer literate workers
TB Tech<br><br>Catalani et al (2014) [30]	Mixed methods human-centred design	Kenya<br><br>Tuberculosis & HIV<br><br>Clinicians	-Patient-specific decision support for provider action, education and behaviour change.<br>-Integrated paper-based processes with electronic medical record.<br>-Supported with educational and motivational information for HCW<br>-Additional inputs including facility staffing and hardware upgrades, supply chain management and provision of mobile radiology units and educational campaign for providers	-Develop, design and pilot test TBTech using a human-centred design approach	-Positive disposition of clinicians to medical record and decision support system<br>-Concerns regarding accuracy and actionability of recommendations<br>-Health system challenges such as unavailability of tests, low staffing and supplies resulted in delayed action and some unactionable recommendations.

txt2MEDLINE<br><br>Armstrong et al (2012) [31]	Pre-post utility evaluation	Botswana<br><br>Multiple domains<br><br>Clinicians of varying cadres	-Mobile phone-based two-way Short Messaging Service (SMS) of clinical guidelines, with MEDLINE query function.	-Usability and usefulness of intervention	-Although pre-intervention study recorded high intention to use, this declined during the one-month trial
ALMANACH<br><br>Shao et al (2015a, 2015b) [32,33]	Controlled non-inferiority trial and qualitative study	Tanzania<br><br>Childhood Illnesses<br><br>Clinicians	-Smartphone or tablet<br>-Electronic medical record system with modified version of IMCI algorithm<br>-Supported by point-of-care tests and simple clinical assessments	-Primary outcomes: proportion of children cured at day 7 and proportion of children who received antibiotics on day zero<br>-Secondary outcomes: proportion of children who were admitted secondarily or who died, and proportion of children who received antibiotics during the study period<br>-Barriers and facilitators to uptake of algorithm and differences between tablets and phones	-Antibiotic prescription reduced by 80% and study reported better clinical outcomes due to increased compliance to guidelines compared to usual practice<br>-Ease and comfort of use, with sustained rational judgment despite certain recommendations by the system<br>-HCWs reported that it made their work efficient and effective compared to usual practice, but raised concerns about increased consultation time and lack of financial incentives for using the service<br>-Patients trust in service delivery improved, although health system constraints hindered completion of the actions and was demotivating

eIMCI<br><br>Mitchell et al (2012); Mitchell et al (2013); DeRenzi et al (2008) [34-36]	Mixed methods before-after cluster trial	Tanzania<br><br>Childhood Illnesses<br><br>Health care professionals	-Personal Digital Assistant with guided decision support using Electronic IMCI protocols for stepwise examination, diagnosis and management.<br>-Algorithm included prompts based on data input.<br>-Language support in English and Swahili	-Perceptions of HCW and caretakers to eIMCI compared to standard paper formats<br>-Adherence to eIMCI guidelines compared to paper formats<br>-Impact of mHealth on quality of IMCI implementation (measured as complete assessment on 15 critical items of the IMCI)	-HCWs were positively predisposed to using the eIMCI; finding it faster and easier, and appreciating the stepwise guidance<br>-Caretakers reported improved assessment of their children and perceived that the eIMCI enhanced provider knowledge and skill<br>-Increased trust and confidence of caretakers in care provided.<br>-Some HCWs reported challenges with eIMCI recommendations that contradicted their preferred course of management<br>-Protocol adherence using eIMCI showed statistically significant improvement from 61%-98% in paper format, to 92%-100%.<br>-Completeness of assessment improved from 21% in the paper format to 71%, and was consistent across study clinics.<br>-Consultation time was not significantly different between the paper system (8.98minutes) and the eIMCI (9.06minutes)
Text Messaging of Malaria Guidelines<br><br>Jones et al (2012); Zurovac et al (2012); Zurovac et al (2011) [37-39]	Cluster randomised controlled trial	Kenya<br><br>Malaria	-One-way SMS guidelines for outpatient management of malaria, supported by unique motivational messages<br>-English Language	-HCW perceptions, experiences and drivers of change of intervention<br>-Improved and maintained adherence guidelines for outpatient paediatric malaria<br>-Correct management with	-Improvement in correct management (24%), which was sustained (25%) up to 6months later<br>-Major improvements in tasks related to dispensing and counselling<br>-HCW responded to the intervention with enthusiasm, perceiving it as innovative, relevant and useful

		Health workers		artemether-lumefantrine and effective counselling	-Most HCW were happy with content, frequency and timing of messages with few concerns about repetition and monotony<br>-Intervention cost amounted to about US $19,000, most (45%) of which was for development and pre-testing. Cost per additional child correctly managed was US $0.50
QUALMAT (Quality of Maternal and Prenatal Care)<br><br>Blank et al (2013); Dalaba et al (2014); Dalaba et al (2015); Mensah et al (2015); Saronga et al (2015); Zakane et al (2014); Duysburgh et al (2016) [40-46]	Mixed methods quasi-experimental study	Tanzania, Ghana and Burkina Faso<br><br>Maternal and Prenatal Health<br><br>Health professionals (non-physicians)	-Guided computer-based decision support system integrated with patient data and algorithmic function for care management (antenatal till early postnatal care) and monitoring.<br>-Includes educational training materials for health workers and an electronic partograph.<br>-Additional intervention components include solar power, regular technical supervision, and performance-based incentives (PBI)	-Usability, acceptance and impact study<br>-Assessment of effect on workflow<br>-Improved quality of care through improved competence and motivation of HCW	*General Findings*<br>-No clear difference between pre- and post-intervention quality scores and scores at non-intervention facilities.<br>-Some variables were inconsistently statistically significant (e.g. in only one study arm, being pre- or post-intervention or intervention, non-intervention)<br>-Post-intervention history taking, monitoring of mother, total technical and inter-personal performance scores were significantly better but remained unsatisfactory<br>- Only care and examination of the newborn scored significantly better between intervention and non-intervention quality scores<br>-Use of mCDSS was acceptable and feasible<br>-Combining mCDSS and PBI did not improve quality of antenatal care<br><br>*Ghana*<br>-Decreased proportion of delivery complications (from 10.7% to 9.6%)

			deaths (from 4 to 1).<br>-48% of financial implementation cost (apprx. US $23,000) was for pre-intervention expenses<br>-No significant increase in consultation time for ANC compared to control sites<br><br>*Tanzania*<br>-Total financial cost of implementation was about US $185,000, 77% of which covered pre-intervention expenses.<br>-No significant increase in consultation time for ANC in intervention sites compared to control sites<br><br>*Burkina Faso*<br>-HCW received mCDSS enthusiastically, expecting it to improve skill and over-reliance on referring patients.<br>-Providers resisted use due to perceived increased workload and complexity of using the system

[a]mCDSS: mobile clinical decision support system.
[b]Although the m4Change study also used the CommCare app, we decided to treat them as independent studies because the interventions were only similar on a technical level and not part of an integrated multicountry study.

Abbreviations
ANC- Antenatal Care
CHEWs- Community Health Extension Workers
CHWs- Community Health Workers
HCWs- Health care Workers
IMCI- Integrated Management of Childhood Illness
mCDSS- mobile clinical decision support system
PHCs- Primary Healthcare Centre's
